# Supplementary material for: Sardine: A modular framework for developing data acquisition and near real-time analysis applications
Source: PLoS One. 2026 Feb 23;21(2):e0330591. doi: 10.1371/journal.pone.0330591 (PMC12928588; doi:10.1371/journal.pone.0330591)
Supplement: Attachment — Submitted filename: Sardine - Response to Reviewers.pdf [file pone.0330591.s002.pdf]

Thank you for the positive and constructive feedback provided on our paper. Below we give point by point responses to the editorial and reviewers requests.

Journal requirements:

When submitting your revision, we need you to address these additional requirements.

1. Please ensure that your manuscript meets PLOS ONE's style requirements, including those for file naming. The PLOS ONE style templates can be found at

https://journals.plos.org/plosone/s/file?id=wjVg/PLOSOne_formatting_sample_main_body.pdf and

https://journals.plos.org/plosone/s/file?id=ba62/PLOSOne_formatting_sample_title_authors_affiliations.pdf

We have verified that the manuscript and file names adhere to PLOS ONE's style guidelines.

2. Thank you for stating the following financial disclosure:

"This work was financed by funding to Michael Orger's lab from the European Research Council (Consolidator Grant, Neurofish-DLV-773012), the Champalimaud Foundation, the Volkswagen Stiftung Life? Initiative (A126151) and through national funds from the Portuguese FCT - Foundation for Science and Technology, I.P., under the projects PTDC/NEU-SCC/5221/2014 and 2023.14873.PEX. A. Lucas Martins received funding from the Portuguese Fundação para a Ciência e Tecnologia (FCT) through the PhD fellowships SFRH/BD/129843/2017 and COVID/BD/152726/2022."

Please state what role the funders took in the study. If the funders had no role, please state: "The funders had no role in study design, data collection and analysis, decision to publish, or preparation of the manuscript."

If this statement is not correct you must amend it as needed.

Please include this amended Role of Funder statement in your cover letter; we will change the online submission form on your behalf.

We have provided the statement above in the cover letter.

If the reviewer comments include a recommendation to cite specific previously published works, please review and evaluate these publications to determine whether they are relevant and should be cited. There is no requirement to cite these works unless the editor has indicated otherwise.

Please review your reference list to ensure that it is complete and correct. If you have cited papers that have been retracted, please include the rationale for doing so in the manuscript text, or remove these references and replace them with relevant current references. Any changes to

the reference list should be mentioned in the rebuttal letter that accompanies your revised manuscript. If you need to cite a retracted article, indicate the article's retracted status in the References list and also include a citation and full reference for the retraction notice.

We have not, to our knowledge, cited a retracted study, or any new citations requested by the reviewers.

This manuscript presents Sardine, an open-source modular framework developed in the .NET environment to support hardware control, data acquisition, and near real-time analysis in experimental sciences, with particular emphasis on neuroscience applications. The framework is motivated by well-recognized challenges in the field, namely the unsustainability of custom-built experimental systems, the difficulty of reusing them across laboratories, and the lack of scalable, fault-tolerant, and domain-agnostic solutions. The authors describe a two-layer modular architecture, provide examples of its application in zebrafish posture tracking and light-sheet microscopy, and compare Sardine with existing frameworks such as Bonsai, LabVIEW, µManager, and Stytra. The manuscript is technically sound, well structured, and offers both conceptual insights and practical demonstrations, making it a valuable contribution to the communities of software engineering and experimental neuroscience.

We sincerely thank the editor for handling our manuscript and for the constructive and detailed feedback from the reviewers. We are very happy that our work was considered to make a valuable contribution to the fields of software engineering and experimental neuroscience and to be technically sound.

While the manuscript is of high quality, there remain some areas where clarification and refinement would strengthen its contribution. A recurring theme across the reviews is the need for quantitative performance benchmarks. Including latency distributions, throughput, CPU and memory usage, and error rates, ideally in a comparative table with other frameworks such as Bonsai or LabVIEW, would provide concrete evidence of Sardine's efficiency and robustness.

Thank you for this feedback. Although our arguments for using Sardine are based predominantly on available features rather than performance alone, we understand that it is important to show that performance is at least as good or better than other state of the art methods. We have run performance benchmarks on the framework, providing quantitative measurements of the latency distribution and the overhead of Sardine during a test operation under different CPU loads, increasing the stress to measure a failure point. We compared Sardine with one of the most comparable open-source frameworks, Bonsai, and showed that Sardine compares very favorably, showing considerably lower overhead in this test. The results were compiled and added to the manuscript in a new section called "Performance benchmarking".

Similarly, while the dual-layer architecture is central to Sardine's design, its novelty and advantages could be emphasized more clearly in both the introduction and the discussion.

We added the following paragraph to the introduction, reinforcing the main advantage of running a dual-layer architecture: "Components are connected in a novel two-layer

network represented by two graphs: one defining hierarchical dependencies and the other defining data flows. By separating connections into critical dependencies and non-critical messaging, a system built with Sardine can operate even in the presence of perturbations, as malfunctioning components and their hierarchical dependencies can be safely terminated by the framework.”

We added the following sentence to the first paragraph of the discussion, emphasizing the main advantage of the two-layer architecture: “, and showed how Sardine's two-layered component aggregator is used to provide fault-tolerance by asserting the working state of critical components.”

In particular, the differences in long-term maintainability and scalability compared with Bonsai’s asynchronous model and LabVIEW’s tightly coupled approach deserve fuller elaboration.

Sardine and Bonsai follow similar approaches to asynchronicity, which should not affect maintainability. We clarified the difference in the asynchronous patterns used by Bonsai and Sardine: “Bonsai, like Sardine, achieves asynchronicity through an event-driven approach. Bonsai leverages the Rx.NET framework, which is built to manipulate live data streams, and Sardine uses a lightweight implementation of a publisher-subscriber pattern.”, and added the note “Furthermore, Bonsai nodes can be built and maintained separately, simplifying code reuse.”. We also added a reference to the Rx.NET framework website (Microsoft. ReactiveX, 2011. Available in https://reactivex.io/).

We added a note reinforcing how Sardine’s modular structure increases its long-term maintainability and scalability: “ This contrasts with the modular structure of Sardine, where each component can be modeled and debugged independently, contributing to long-term maintainability and scalability of the system”.

Another important point relates to cross-platform usability. At present, the GUI is Windows-specific through .NET/WPF. The manuscript would benefit from further discussion of how Sardine can be used by non-Windows users, for example through command-line operation, headless mode, integration with Python interfaces, or containerization solutions such as Docker. A brief roadmap outlining possible directions for cross-platform support would broaden the appeal of the framework considerably.

Since the core of Sardine is written in .NET 8, which already has native cross-platform support, any non-Windows user can already use the Sardine core architecture. In the discussion, we suggest steps that any user with sufficient interest can take to produce compatible GUIs to use Sardine in non-Windows environments: “Recent cross-platform alternatives, such as AvaloniaUI or newer .NET frameworks like .NET MAUI could replace WPF, allowing built-in Sardine GUI to be available on most operating systems”.

Some technical aspects would also benefit from additional detail. The description of vessel abstractions and state management is clear, but more information is needed on how Sardine

handles potential race conditions when multiple vessels attempt to access a shared resource, and what the performance overhead is of vessel creation and dynamic reloading in high-throughput experiments.

We have added a description on how multiple accesses to a shared resource are resolved to the manuscript: "Furthermore, since messages are placed in the inbox queue before processing, if multiple vessels try to communicate with the same downstream node, their messages will be added to the queue and processed sequentially, avoiding collisions in accessing reserved resources. Furthermore, since all messages carry a unique ID, messages can be uniquely resolved and ordered by the user as appropriate if such behavior is required".

Since vessel creation and dynamic reloading are not expected to occur at high rates during the experiment, the practical performance cost of these actions should be negligible, as any temporary delays will be dampened by the modules' inbox queues. We have added a note describing this design consideration to the manuscript: "The inbox queue also dampens any temporary delays caused by vessel creation or their dynamic reloading."

Similarly, although the examples provided are compelling, the results could be expanded to include quantitative performance outcomes for zebrafish posture tracking and microscopy applications, together with descriptions of how the system performs under stress conditions such as heavy CPU load or unexpected crashes.

In-depth performance tests for concrete applications, which depend on specific hardware parts such as cameras or data acquisition systems, may not represent the framework accurately; therefore, we have designed quantitative benchmarks, described above, that can demonstrate performance in a more neutral setting. Nevertheless, we added information on the average time required for our machine to compute fish posture per camera frame to the "Use Cases" section of the manuscript: "This simple tracker is efficient, computing tail posture in under 150 microseconds per frame, and making it robust to other stresses on system performance".

We have added a description of the framework performance under stress conditions and failure modes in the new manuscript section "Performance benchmarks".

Accessibility is another important factor. While the manuscript notes that Sardine is hosted on GitHub, providing readers with simple demo scripts or tutorials that can be run without specialized hardware would make it easier for the community to engage with and adopt the framework.

To enhance accessibility, we have made a demo software for an online zebrafish tail tracker, which does not require specialized hardware but instead uses a fish tail recording that is replayed by a virtual camera component, and is publicly available on GitHub (https://github.com/orger-lab/sardine-components). It is accompanied by a

step-by-step tutorial on how to install and run the demo, and a link to this repository is available in the Sardine readme.

At the same time, future plans for improving user experience and expanding multilingual support (for example in Spanish, Turkish, or Chinese) would help to establish Sardine as a globally relevant tool.

As we do not have the knowledge to properly translate or validate translations into most languages, we believe that our providing multilingual translations of the Sardine documentation would not effectively expand its reach. Moreover, due to the ease of modern translation tools, such as those enabled by large language models, it should be possible for any user to generate their own translation by copy-pasting the documentation into such a tool (e.g. ChatGPT) to translate into their own language. Furthermore, as an open-source framework, any interested user could modify Sardine and add their own translation to the framework.

In terms of presentation, the manuscript would benefit from modest shortening to maintain reader engagement.

We appreciate the suggestion and have made efforts to edit the language of the manuscript to make it more concise. To keep the manuscript size manageable, even in light of the proposed clarifications and additions, we have substantially reduced the length of the introduction section, removing some redundancy, while maintaining the more important explanations of the methods and benchmarking.

Certain terms such as "freightering" and "invalidator" could be replaced with more standard software engineering terminology.

We replaced references to "freighting" in the manuscript by an explanation that the Freighter class is responsible for instantiating Vessels. We kept "invalidation" as we believe it correctly portrays the meaning of rendering an object as invalid/needing an update, and it is a term that sees use in other software engineering applications (e.g. UI element invalidation, cache invalidation). To clarify this meaning, we adapted the manuscript with the following sentence: "The invalidator properly marks the component as invalid in cases of termination or failure".

Figures 2–4, although informative, appear dense and of low resolution; clearer rendering, more intuitive labeling, and color coding would help make vessel states and data graphs easier to follow.

We have adapted the colors of the vessel diagrams and data graphs to make the text easier to read and follow. We have also re-rendered all figures with improved resolution.

Finally, care should be taken to ensure that all acronyms (such as WPF and sCMOS) are defined at first mention.

The acronyms WPF, UI, GUI, and sCMOS are now defined at first mention. The acronym ROI was replaced by its definition.

In summary, the manuscript is a significant and timely contribution that successfully addresses challenges in developing modular, scalable, and reusable frameworks for experimental research. The work demonstrates both technical rigor and practical relevance. With the inclusion of benchmarking data, clarification of architectural novelty, improved guidance on cross-platform use, and modest improvements to figures and presentation, the manuscript will be ready for publication.

We have carefully addressed all of the editor and reviewers' comments and believe the manuscript has been significantly strengthened as a result of their suggestions, better demonstrating the value of Sardine as a modular framework for developing data acquisition and near real-time analysis applications. In particular, we have added performance benchmark data, explained in the manuscript how Sardine's architecture differs from other state of the art solutions, clarified how cross-platform use is currently being supported by Sardine, and re-rendered all figures at a higher resolution. We believe that the revised manuscript now meets the standards for publication in PLOS ONE.

5. Review Comments to the Author

Reviewer #1: This study introduces Sardine, a software framework developed for use in experimental sciences, particularly in data-intensive and hardware-focused fields such as neuroscience. Sardine was developed within the .NET ecosystem and aims to facilitate the establishment of hardware control and near real-time data processing systems by operating in a modular structure. The software features fault-tolerant architecture, data queue management, module independence, dual-layer module integration, interface creation tools, and remote access. Examples of Sardine's use include neuroscience experiments such as zebrafish tracking and light-transmission microscope control. The work stems from practical problems such as the unsustainability of hardware-software integrations used in experimental research and the difficulty of reusing custom systems. Sardine is a framework developed as a solution to these problems. Its motivation is clear, powerful, and field-based. It combines hardware control with real-time data processing, creates fault-tolerant systems, and offers an open-source platform.
Although software-based, it offers significant methodological contributions to the design of experimental systems in disciplines such as neuroscience. Comparisons with existing solutions (Bonsai, LabVIEW, µManager, etc.) are clear. However, I would like to kindly offer the following suggestions to the authors:

We thank the reviewer for their careful reading and comments on the manuscript, and for their positive assessment of its contribution to the design of experimental systems. We have endeavoured to take account of all of their suggestions as detailed below.

1- Benchmarking is lacking. No direct performance comparison has been made with alternatives such as Bonsai or LabVIEW. A Benchmarking Table is recommended, and Sardine's comparative performance data with other systems should be used.

Thank you for the feedback. While our arguments for using Sardine are based predominantly on available features rather than performance alone, we do understand that it is important to show that performance is at least as good or better than other state of the art methods. We have run performance benchmarks on the framework, providing quantitative measurements of the latency distribution and the overhead of Sardine during a test operation under different CPU loads, increasing the stress to measure a failure point. We compared Sardine with one of the most comparable open-source frameworks, Bonsai, and showed that Sardine compares very favorably, showing considerably lower overhead in this test. The results were compiled and added to the manuscript in a new section called "Performance benchmarking".

2- Evaluation of user experience/learning curve is limited and should be provided in detail. Additionally, further content could be provided on advanced customizations in GUI design.

While we believe that providing comprehensive user experience evaluations is outside the scope of this work, we have allowed naive users to build the example and test applications available on the GitHub repository, following the provided tutorials, who have been able to fully run the examples with no difficulties, and we have incorporated their feedback in preparing the documentation and demo. Regarding advanced GUI designs and their customizations, users are encouraged to use any WPF tools to enhance their own GUIs. We have added a sentence reinforcing this to the discussion section of the manuscript: "Users can further customize their GUIs by using any natively available WPF resource or UI control, as well as any of the many third-party libraries available online.".

3- Real-time test results could be more detailed (e.g., CPU load, crash scenarios, resource usage).

We have added, to the new section "Performance benchmarks" described previously, data on the performance of Sardine, including the overhead of the framework under different CPU loads. In that section, we also describe the results of crashes caused by system overload. Furthermore, we added information on the average time required for our machine to compute fish posture per camera frame to the "Use Cases" section of the manuscript: "This simple tracker is efficient, computing tail posture in under 150 microseconds per frame, and making it robust to other stresses on system performance".

Also, a plan or roadmap should be shared for Docker/Cross-platform support (currently limited to .NET/WPF).

Since the core of Sardine is written in .NET 8, which already has native cross-platform support, any non-Windows user can already use the Sardine core architecture. In the discussion, we suggest steps that any user with sufficient interest can take to produce compatible GUIs to use Sardine in non-Windows environments: "Recent cross-platform

alternatives, such as AvaloniaUI or newer .NET frameworks like .NET MAUI could replace WPF, allowing built-in Sardine GUI to be available on most operating systems".

4- Support for multiple languages could be added; common languages should be added (e.g., Spanish, Turkish, and Chinese). I recommend adding them for global distribution.

As we do not have the knowledge to properly translate or validate translations into most languages, we believe that our providing multilingual translations of the Sardine documentation would not effectively expand its reach. Moreover, due to the ease of modern translation tools, such as those enabled by large language models, it should be possible for any user to generate their own translation by copy-pasting the documentation into such a tool (e.g. ChatGPT) to translate into their own language. Furthermore, as an open-source framework, any interested user could modify Sardine and add their own translation to the framework.

My polite suggestion is to accept with minor revisions.

We are grateful to Reviewer #1 for recognizing Sardine's motivation and contributions, and for providing valuable suggestions that have strengthened our work, particularly the recommendation for performance benchmarking, which has significantly improved the manuscript.

Reviewer #2: In the manuscript entitled "Sardine: a modular framework for developing data acquisition and near real-time analysis applications" authors presented the detailed structure and execution of Sardine open-source modular software built in .NET environment, for controlling experimental setups and real-time data acquisition/analysis. The manuscript is technically sound and provides both conceptual and practical insights into the framework. It is a well structured presentation with detailed examples and comparative analysis with alternative solutions. Overall, this is a valuable contribution to the field of software engineering, with potential broader applications. However, still there is a scope for further improvement by addressing the following suggestions and comments to improve clarity and readability.

We appreciate the time taken by this reviewer to carefully read and provide feedback on the manuscript. We are glad they find it to be technically sound and making a valuable contribution to the field. We are very grateful for the helpful suggestions and we detail below how we have incorporated this feedback to improve the manuscript.

1. Through the developed software Sardine provides alternate solution to the existing frameworks (e.g., Bonsai, LabVIEW, µManager, Stytra), the novelty of Sardine's "two-layer architecture" (link layer + data layer) may be more explicitly emphasized in the Introduction and Discussion.

Thank you for this suggestion. We added the following paragraph to the introduction, reinforcing the main advantage of running a dual-layer architecture: "Components are connected in a novel two-layer network represented by two graphs: one defining hierarchical dependencies and the other defining data flows. By separating connections

into critical dependencies and non-critical messaging, a system built with Sardine can operate even in the presence of perturbations, as malfunctioning components and their hierarchical dependencies can be safely terminated by the framework."

We added the following sentence to the first paragraph of the discussion: ", and showed how Sardine's two-layered component aggregator is used to provide fault-tolerance by asserting the working state of critical components."

2. Authors may clarify how Sardine differs with respect to Bonsai's asynchronous model and LabVIEW's coupling in terms of long-term maintainability and scalability (other than error handling).

Sardine and Bonsai follow similar approaches to asynchronicity, which should not affect maintainability. We clarified the difference in the asynchronous patterns used by Bonsai and Sardine: "Bonsai, like Sardine, achieves asynchronicity through an event-driven approach. Bonsai leverages the Rx.NET framework, which is built to manipulate live data streams, and Sardine uses a lightweight implementation of a publisher-subscriber pattern.", and added the note "Furthermore, Bonsai nodes can be built and maintained separately, simplifying code reuse."

We added a note reinforcing how Sardine's modular structure increases its long-term maintainability and scalability: " This contrasts with the modular structure of Sardine, where each component can be modeled and debugged independently, contributing to long-term maintainability and scalability of the system".

3. Currently, Sardine GUIs limits usage on Windows only. Authors may describe how non-Windows users can still utilize Sardine (e.g., CLI-only operation, headless mode, or integration with Python frontends).

Since the core of Sardine is written in .NET 8, which already has native cross-platform support, any non-Windows user can already use the Sardine core architecture. In the discussion, we suggest steps that any user with sufficient interest can take to produce compatible GUIs to use Sardine in non-Windows environments: "Recent cross-platform alternatives, such as AvaloniaUI or newer .NET frameworks like .NET MAUI could replace WPF, allowing built-in Sardine GUIs to be available on most operating systems".

4. The vessel abstraction and state management (IsOnline, IsActive, ExecuteCall) are clearly explained, but some technical details require more elaboration. For example:
• How does Sardine handle race conditions when multiple vessels try to access a shared resource?
• What is the overhead of vessel creation and dynamic reloading in high-throughput experiments?

We have added a description on how multiple accesses to a shared resource are resolved to the manuscript: “Furthermore, since messages are placed in the inbox queue before processing, if multiple vessels try to communicate with the same downstream node, their messages will be added to the queue and processed sequentially, avoiding collisions in accessing reserved resources. Furthermore, since all messages carry a unique ID, messages can be uniquely resolved and ordered as appropriate if such behavior is required”.

Since vessel creation and dynamic reloading are not expected to occur at high rates during the experiment, the practical performance cost of these actions should be negligible, as any temporary delays will be dampened by the modules' inbox queues. We have added a note describing this design consideration to the manuscript: “The inbox queue also dampens any temporary delays caused by vessel creation or their dynamic reloading.”

5. Though the availability of Sardine on GitHub repositories is mentioned, authors may provide some working examples (e.g., a reproducible demo script or tutorial) in the manuscript that allows readers to test Sardine immediately without specialized hardware.

We made a demo software for an online zebrafish tail tracker, which does not require specialized hardware but instead uses a fish tail recording that is replayed by a virtual camera component, and is publicly available on GitHub (https://github.com/orger-lab/sardine-components). It is accompanied by a step-by-step tutorial on how to install and run the demo, and a link to this repository is available in the Sardine readme.

6. While the zebrafish posture tracking and light-sheet microscopy examples are appealing, quantitative performance metrics may be provided for posture tracking, report actual latency distributions, error rates, and system throughput.

To show the efficiency and robustness of Sardine, we ran performance benchmarks to the framework, providing quantitative measurements of the latency distribution and the overhead of Sardine while running a test operation under distinct CPU loads. The results were compiled and added to the manuscript to a new section called “Performance benchmarking”. We have also added information regarding the posture tracking code, showing that it executes very rapidly, which, combined with Sardines low overhead, make it robust to additional stresses on resources.

Minor Comments:

1. The current manuscript is a valuable contribution but the length of the manuscript diverts the readers interest. Authors are advised to shorten the manuscript with more focus to attach the readers interest.

We appreciate the suggestion and have made efforts to edit the language of the manuscript to make it more concise. To keep the manuscript size manageable, while including also the proposed clarifications and additions, we have reworked the

introduction section to make it substantially shorter and less redundant.

2. Usage of some words like "freightering", "invalidator" may be replaced with more standard terminology.

We replaced references to “freighting” in the manuscript by an explanation that the Freighter class is responsible for instantiating Vessels. We kept “invalidation” as we believe it correctly portrays the meaning of rendering an object as invalid/needing an update, and it is a term that sees use in other software engineering applications (e.g. UI element invalidation, cache invalidation). To clarify this meaning, we adapted the manuscript with the following sentence: “The invalidator properly marks the component as invalid in cases of termination or failure”.

3. Figures 2–4 are informative but somewhat dense and with low resolution. Use clearer color coding or labels to make vessel states and data/link graphs more intuitive.

We have adapted the colors of the vessel diagrams and data graphs to make the text easier to read and follow. We have also re-rendered all figures at a higher resolution.

4. Ensure all acronyms (e.g., WPF, sCMOS) are defined at first use.

We edited the manuscript so that the acronyms WPF, UI, GUI, and sCMOS are defined at first mention. The acronym ROI was replaced by its definition.

We are grateful to Reviewer #2 for their careful and positive evaluation and constructive feedback, which has helped us address important technical details and improve the readability of the manuscript.

Reviewer #3: The manuscript provides valuable contributions to both the software engineering and experimental neuroscience communities by offering a domain-agnostic, modular, and scalable solution for real-time data acquisition and analysis. To further strengthen the article, the authors could include quantitative performance benchmarks (e.g., latency, memory usage, CPU load) in the Methods or Use Cases sections, provide diagrams or figures for all architectural components, and emphasize cross-platform GUI alternatives for broader applicability.

We are grateful to Reviewer #3 for their careful review of our manuscript and we are happy that they found that it makes a valuable contribution in its field. We agree with the point raised about performance benchmarks, and have added these to the paper, as detailed below, as well as addressing other suggestions.

The research article presents a well-structured, comprehensive, and technically rigorous description of a software framework designed for complex experimental workflows. The manuscript effectively explains the architecture of Sardine, highlighting its modularity, asynchronous communication between components, service-based flexibility, and user-friendly desktop application support. The inclusion of real-world use cases, such as the high-speed zebrafish tracking system and the light-sheet microscopy system, demonstrates the framework’s robustness, high performance, and versatility in handling demanding data acquisition tasks. The

comparison with existing general-purpose and domain-specific solutions is insightful, clearly positioning Sardine within the landscape of scientific software frameworks. The discussion also acknowledges current limitations, such as the Windows-specific UI implementation and the need for performance evaluation in long-term applications, which reflects a transparent and thoughtful
analysis by the authors.

Abstract:

The abstract effectively introduces Sardine as a modular, .NET-based framework designed for developing data acquisition and near real-time analysis applications. It concisely highlights the key features of the framework, including modularity, asynchronous communication, and GUI support, providing readers with a clear overview of the system's purpose and capabilities. This sets an appropriate context for the detailed discussion in the rest of the article.

Introduction:

The introduction provides a strong rationale for the development of Sardine, situating it within the landscape of experimental software tools. By comparing Sardine conceptually to existing frameworks such as Bonsai, LabVIEW, and μManager, the authors highlight the specific challenges Sardine addresses and the potential benefits of adopting a modular and flexible approach. This section effectively motivates the need for the framework and frames its unique contributions.

Methods:

The functional organization of Sardine: This section clearly explains the fleet and vessel architecture, presenting a well-structured view of how components are organized and interact within the system. The explanation is detailed enough to give readers confidence in understanding Sardine's core design. Vessels communicate asynchronously through messages: The mechanism of message-passing is articulated clearly, showing how asynchronous communication is implemented and its benefits in decoupling components. Services provide extra flexibility to Sardine: The service abstraction is well-explained, including how services are sequentially accessed and used to extend the functionality of the system without blocking critical operations. Sardine produces user-friendly desktop applications: The integration with WPF and the vessel- based UI is described effectively, demonstrating how Sardine simplifies the creation of responsive, reactive desktop applications for complex workflows.

Operation:

Creating a Fleet and encapsulating components in Vessels using the Freighter: This section provides a practical coding example along with a step-by-step diagram, making it easy to follow how components are encapsulated and managed within a fleet. Adding a data operation: The explanation of Source, Transformer, and Sink patterns is clear, showing how data flows are constructed and processed within the Sardine framework.

Building a desktop application:

Adding a GUI: The discussion on VesselUserControl integration and reactive UI binding

effectively demonstrates how user interfaces can be linked to underlying components with minimal boilerplate code. Adding a service: The example highlights the interaction between vessels and services, illustrating how services can enhance functionality and communicate with multiple components.

Layout and control of a Sardine application:
Using the settings manager: The use of SARDINE.xml for configuration is well-described, showing how settings can be fetched and applied to dynamically control system behavior. Using the logger: Logging capabilities, including message levels and capturing output from components, are thoroughly explained, emphasizing Sardine's robustness in monitoring Operations. Extending the Sardine application through UI helpers: The framework's extensibility through reflection is well-demonstrated, highlighting the ease of adding custom UI elements and features.

Use cases:
Online tracking system for head-fixed larval zebrafish: This use case is concrete and detailed, including workflow steps, performance metrics, and validation of the framework's suitability for high-speed, real-time data processing. Custom-built light-sheet microscopy system: Demonstrates Sardine's capacity to orchestrate complex, highly coupled hardware components, emphasizing the framework's flexibility in managing experimental setups. General-purpose graph-based systems: The comparison with frameworks such as Bonsai, LabVIEW, Heron, Falcon, and BRAND is thorough, highlighting differences in communication models, synchronization, and suitability for different use cases. Domain-specific solutions: µManager, Stytra, and improv are discussed, showing Sardine's advantages in flexibility and integration compared to domain-specific tools.

Discussion:
The discussion clearly articulates Sardine's contributions, emphasizing modularity, reusability, and maintainability over long-term use. The authors thoughtfully acknowledge current limitations, such as Windows-specific UI constraints and potential performance optimizations, while suggesting improvements like cross-platform GUI support and the use of circular buffers. Overall, the discussion effectively positions Sardine as a versatile and extensible framework capable of addressing diverse experimental software needs.

Recommendation:
I strongly recommend the publication of this article. The manuscript provides valuable contributions to both the software engineering and experimental neuroscience communities by offering a domain-agnostic, modular, and scalable solution for real-time data acquisition and analysis. To further strengthen the article, the authors could include quantitative performance benchmarks (e.g., latency, memory usage, CPU load) in the Methods or Use Cases sections,

Thank you for this feedback. Although our arguments for using Sardine are based predominantly on available features rather than performance alone, we understand that it is important to show that performance is at least as good or better than other state of the

art methods. We have run performance benchmarks on the framework, providing quantitative measurements of the latency distribution and the overhead of Sardine during a test operation under different CPU loads, increasing the stress to measure a failure point. We compared Sardine with one of the most comparable open-source frameworks, Bonsai, and showed that Sardine compares very favorably, showing considerably lower overhead in this test. The results were compiled and added to the manuscript in a new section called “Performance benchmarking”.

provide diagrams or figures for all architectural components,

We have endeavoured to improve the presentation of the framework components in the existing figures. Figure 2 explains the architecture of the Vessel, and Figure 3 represents the two-layer architecture of Sardine, the main components of the framework. We adapted the color scheme of both Figures 2 and 3 to enhance clarity. Given the suggestions to improve readability and to create a shorter and more focused manuscript, we believe that adding further diagrams or figures detailing the internals of the framework would not be helpful.

and emphasize cross-platform GUI alternatives for broader applicability.

Since the core of Sardine is written in .NET 8, which already has native cross-platform support, any non-Windows user can already use the Sardine core architecture. In the discussion, we have suggested steps that any user with sufficient interest can take to produce compatible GUIs to use Sardine in non-Windows environments: “Recent cross-platform alternatives, such as AvaloniaUI or newer .NET frameworks like .NET MAUI could replace WPF, allowing built-in Sardine GUI to be available on most operating systems”.

Final Observation:
Overall, Sardine represents a significant advancement in modular software frameworks for scientific research. Its design promotes code reuse, simplifies complex hardware orchestration, and supports high-throughput data processing. If widely adopted and maintained, it has the potential to become a standard tool for laboratories requiring flexible, high-performance, and user-friendly experimental software solutions. The framework’s open-source nature also encourages community-driven development, ensuring continuous improvement and adaptation to new experimental paradigms.

We thank Reviewer #3 for their careful review, constructive suggestions which have helped us to improve the manuscript, and their recommendation for publication.
