# Supplementary material for: Sardine: A modular framework for developing data acquisition and near real-time analysis applications
Source: PLoS One. 2026 Feb 23;21(2):e0330591. doi: 10.1371/journal.pone.0330591 (PMC12928588; doi:10.1371/journal.pone.0330591)
Supplement: Attachment — Submitted filename: Review Report.docx [file pone.0330591.s001.docx]

**Review Report**

**Authors	:** A. Lucas Martins, Alexandre Laborde, Michael Orger

**Title	:** Sardine: a modular framework for developing data acquisition and near real-

time analysis applications

The research article presents a well-structured, comprehensive, and technically rigorous description of a software framework designed for complex experimental workflows. The manuscript effectively explains the architecture of Sardine, highlighting its modularity, asynchronous communication between components, service-based flexibility, and user-friendly desktop application support. The inclusion of real-world use cases, such as the high-speed zebrafish tracking system and the light-sheet microscopy system, demonstrates the framework’s robustness, high performance, and versatility in handling demanding data acquisition tasks. The comparison with existing general-purpose and domain-specific solutions is insightful, clearly positioning Sardine within the landscape of scientific software frameworks. The discussion also acknowledges current limitations, such as the Windows-specific UI implementation and the need for performance evaluation in long-term applications, which reflects a transparent and thoughtful analysis by the authors.

**Abstract:**

The abstract effectively introduces Sardine as a modular, .NET-based framework designed for developing data acquisition and near real-time analysis applications. It concisely highlights the key features of the framework, including modularity, asynchronous communication, and GUI support, providing readers with a clear overview of the system’s purpose and capabilities. This sets an appropriate context for the detailed discussion in the rest of the article.

**Introduction:**

The introduction provides a strong rationale for the development of Sardine, situating it within the landscape of experimental software tools. By comparing Sardine conceptually to existing frameworks such as Bonsai, LabVIEW, and µManager, the authors highlight the specific challenges Sardine addresses and the potential benefits of adopting a modular and flexible approach. This section effectively motivates the need for the framework and frames its unique contributions.

**Methods:**

The functional organization of Sardine: This section clearly explains the fleet and vessel architecture, presenting a well-structured view of how components are organized and interact within the system. The explanation is detailed enough to give readers confidence in understanding Sardine’s core design.

Vessels communicate asynchronously through messages: The mechanism of message-passing is articulated clearly, showing how asynchronous communication is implemented and its benefits in decoupling components.

Services provide extra flexibility to Sardine: The service abstraction is well-explained, including how services are sequentially accessed and used to extend the functionality of the system without blocking critical operations.

Sardine produces user-friendly desktop applications: The integration with WPF and the vessel-based UI is described effectively, demonstrating how Sardine simplifies the creation of responsive, reactive desktop applications for complex workflows.

**Operation:**

Creating a Fleet and encapsulating components in Vessels using the Freighter: This section provides a practical coding example along with a step-by-step diagram, making it easy to follow how components are encapsulated and managed within a fleet.

Adding a data operation: The explanation of Source, Transformer, and Sink patterns is clear, showing how data flows are constructed and processed within the Sardine framework.

**Building a desktop application:**

Adding a GUI: The discussion on VesselUserControl integration and reactive UI binding effectively demonstrates how user interfaces can be linked to underlying components with minimal boilerplate code.

Adding a service: The example highlights the interaction between vessels and services, illustrating how services can enhance functionality and communicate with multiple components.

**Layout and control of a Sardine application:**

Using the settings manager: The use of SARDINE.xml for configuration is well-described, showing how settings can be fetched and applied to dynamically control system behavior.

Using the logger: Logging capabilities, including message levels and capturing output from components, are thoroughly explained, emphasizing Sardine’s robustness in monitoring operations.

Extending the Sardine application through UI helpers: The framework’s extensibility through reflection is well-demonstrated, highlighting the ease of adding custom UI elements and features.

**Use cases:**

Online tracking system for head-fixed larval zebrafish: This use case is concrete and detailed, including workflow steps, performance metrics, and validation of the framework’s suitability for high-speed, real-time data processing.

Custom-built light-sheet microscopy system: Demonstrates Sardine’s capacity to orchestrate complex, highly coupled hardware components, emphasizing the framework’s flexibility in managing experimental setups.

General-purpose graph-based systems: The comparison with frameworks such as Bonsai, LabVIEW, Heron, Falcon, and BRAND is thorough, highlighting differences in communication models, synchronization, and suitability for different use cases.

Domain-specific solutions: µManager, Stytra, and improv are discussed, showing Sardine’s advantages in flexibility and integration compared to domain-specific tools.

**Discussion:**

The discussion clearly articulates Sardine’s contributions, emphasizing modularity, reusability, and maintainability over long-term use. The authors thoughtfully acknowledge current limitations, such as Windows-specific UI constraints and potential performance optimizations, while suggesting improvements like cross-platform GUI support and the use of circular buffers. Overall, the discussion effectively positions Sardine as a versatile and extensible framework capable of addressing diverse experimental software needs.

**Recommendation:**

I strongly recommend the publication of this article. The manuscript provides valuable contributions to both the software engineering and experimental neuroscience communities by offering a domain-agnostic, modular, and scalable solution for real-time data acquisition and analysis. To further strengthen the article, the authors could include quantitative performance benchmarks (e.g., latency, memory usage, CPU load) in the Methods or Use Cases sections, provide diagrams or figures for all architectural components, and emphasize cross-platform GUI alternatives for broader applicability.

**Final Observation:**

Overall, Sardine represents a significant advancement in modular software frameworks for scientific research. Its design promotes code reuse, simplifies complex hardware orchestration, and supports high-throughput data processing. If widely adopted and maintained, it has the potential to become a standard tool for laboratories requiring flexible, high-performance, and user-friendly experimental software solutions. The framework’s open-source nature also encourages community-driven development, ensuring continuous improvement and adaptation to new experimental paradigms.
