# Supplementary material for: Sardine: A modular framework for developing data acquisition and near real-time analysis applications
Source: PLoS One. 2026 Feb 23;21(2):e0330591. doi: 10.1371/journal.pone.0330591 (PMC12928588; doi:10.1371/journal.pone.0330591)
Supplement: Attachment — Submitted filename: Minor Revision.docx [file pone.0330591.s003.docx]

This manuscript presents *Sardine*, a modular, fault-tolerant framework for near real-time data acquisition and control in experimental systems. The work is technically sound, clearly written, and well motivated. The proposed architecture is thoughtfully designed, and the framework addresses a genuine and widely encountered problem in experimental research software. The examples provided convincingly demonstrate real-world applicability.

I recommend **acceptance with minor revisions**.

**Minor Revision Requests**

- **Clarify Scope and Limitations**
  While the framework is presented as domain-agnostic, all concrete examples are drawn from neuroscience. The authors should briefly clarify the intended scope of applicability and explicitly state any assumptions or constraints (e.g., reliance on desktop OS scheduling, UI platform limitations).
- **Quantitative Performance Context**
  The manuscript reports impressive performance figures in the use cases. Adding a short clarifying paragraph—either in the Methods or Discussion—describing how these figures were measured (e.g., typical hardware, timing methodology) would improve interpretability without requiring additional experiments.
- **Comparison with Existing Frameworks**
  The comparison section is informative but largely qualitative. A short, structured summary (for example, a table or bullet list) highlighting key differences between Sardine and closely related frameworks (e.g., Bonsai, LabVIEW) would help readers quickly contextualize the contribution.
- **Learning Curve and User Onboarding**
  The abstraction model (fleets, vessels, services, dual graphs) is powerful but may present a learning curve for new users. A brief comment on expected onboarding effort, recommended development patterns, or typical usage scenarios would be helpful.
- **UI and Cross-Platform Considerations**
  Since the current GUI implementation relies on WPF, the authors should more explicitly note the Windows-specific nature of the provided UI components and, if appropriate, briefly mention possible future directions for cross-platform interfaces.

**Final Recommendation**

This is a strong and valuable contribution that will be of interest to researchers developing complex experimental systems. The requested revisions are minor and primarily aimed at improving clarity and positioning rather than addressing substantive technical concerns.
